# Efficacy of an mHealth Behavior Change Intervention for Promoting Physical Activity in the Workplace: Randomized Controlled Trial

**DOI:** 10.2196/44108

**Published:** 2023-04-27

**Authors:** Salah Alshagrawi, Syed Taha Abidi

**Affiliations:** 1 Saudi Electronic University Riyadh Saudi Arabia

**Keywords:** text messaging, mobile phones, physical activity, eHealth, pedometer, smartphone, activity tracker, accelerometer, behavioral, workplace, risk factor, noncommunicable disease, BMI, wellness

## Abstract

**Background:**

Insufficient physical activity (PA) is a well-established risk factor for several noncommunicable diseases such as cardiovascular diseases, cancer, diabetes, depression, and dementia. The World Health Organization (WHO) advises that individuals engage in 150 minutes of moderate PA per week or 75 minutes of intense PA per week. According to the WHO’s latest report, 23% of adults fail to meet the minimum recommended level of PA. The percentage was even higher in a recent global study that showed 27% of adults were insufficiently active and reported a 5% increase in the prevalence trend of insufficient PA between 2001 and 2016. The study also showed the rate of insufficient PA among countries varied significantly. For instance, it was estimated that 40% were insufficiently active in the United States, and the percentage was even higher in Saudi Arabia (more than 50%). Governments are actively developing policies and methods to successfully establish a PA-inducing environment that encourages a healthy lifestyle in order to address the global steady decline in PA.

**Objective:**

The purpose of this study was to determine the effectiveness of mobile health (mHealth) interventions, particularly SMS text messaging interventions, to improve PA and decrease BMI in healthy adults in the workplace.

**Methods:**

In this parallel, 2-arm randomized controlled trial, healthy adults (N=327) were randomized to receive an mHealth intervention (tailored text messages combined with self-monitoring (intervention; n=166) or no intervention (control; n=161). Adults who were fully employed in an academic institution and had limited PA during working hours were recruited for the study. Outcomes, such as PA and BMI, were assessed at baseline and 3 months later.

**Results:**

Results showed significant improvement in PA levels (weekly step counts) in the intervention group (β=1097, 95% CI 922-1272, *P*<.001). There was also a significant reduction in BMI (β=0.60, 95% CI 0.50-0.69, *P*<.001).

**Conclusions:**

Combining tailored text messages and self-monitoring interventions to improve PA and lower BMI was significantly effective and has the potential to leverage current methods to improve wellness among the public.

## Introduction

Insufficient physical activity (PA) is a well-established risk factor for several noncommunicable diseases such as cardiovascular diseases, cancer, diabetes, depression, and dementia [1-4]. The World Health Organization (WHO) advises that individuals engage in 150 minutes of moderate PA per week or 75 minutes of intense PA per week [2]. According to the WHO’s latest report, 23% of adults fail to meet the minimum recommended level of PA [5]. The percentage was even higher in a recent global study that showed 27% of adults were insufficiently active and reported a 5% increase in the prevalence trend of insufficient PA between 2001 and 2016 [6]. The study also showed that the rate of insufficient PA among countries varied significantly. For instance, it was estimated that 40% were insufficiently active in the United States, and the percentage was even higher in Saudi Arabia (more than 50%) [6]. Governments are actively developing policies and methods to successfully establish a PA-inducing environment that encourages a healthy lifestyle in order to address the global steady decline in PA.

As a result of economic expansion and technological advancement, more individuals are working in sedentary jobs [1], a significant factor in the prevalence of insufficient PA [7]. On average, about 8.5 hours a day are spent working by employees, and with improvements in automation and information technology use, it is anticipated that occupational sedentary time will rise even higher in the future [7]. Office workers spend up to 71% of their working hours sitting down, and work accounts for around half of their sitting time [8]. A longer duration of time spent sitting is associated with a higher risk of obesity, cancer, diabetes, and all-cause mortality [9-12]. Mobile health (mHealth) applications have the potential to considerably reduce the prevalence of insufficient PA by overcoming various barriers such as the involved cost, lack of motivation and skills, poorly designed and applied interventions, and fewer tools and resources [13,14]. Compared to traditional PA interventions, mHealth PA interventions are estimated to be 12% more effective in increasing the level of PA [11,12]. In the workplace setting, a systematic review of 25 quasi-experimental and experimental studies observed that 56% of studies demonstrated a significant increase in PA [7]. However, other studies reported some limitations related to the type and content of PA interventions [15,16].

Compared to other interventions, tailored interventions and those grounded in theoretical concepts such as the self-determination theory [17], the transtheoretical model [18], the theory of planned behavior [19], and behavior change methods appear to be effective in promoting PA [20-22]. Furthermore, social relationships and social contexts influence and promote healthy behaviors [23,24]. The developments in individually tailored applications seem to be a beneficial component to incorporate into behavioral modification interventions [25].

Due to the rapid development of new generations of a diverse range of sensors and the application of machine learning techniques, previous research examined the potential use of mHealth to improve PA in a variety of groups, including workers [26]. A study on mHealth applications grounded in the self-determination theory found that motivated employees had higher PA levels [27]. Additionally, mHealth interventions can motivate employees to stay committed to their PA goals in addition to tracking their data and achievements [28,29]. In contrast, some studies found no significant differences between groups of employees in terms of PA level [30,31]. Thus, in this study, we aimed to examine the effectiveness of mHealth, specifically text messages combined with self-monitoring, on the level of PA among workers in sedentary jobs.

## Methods

### Study Design

This study was a randomized controlled trial (RCT) to investigate the effect of an mHealth intervention (text messages and self-monitoring) among employees at a large academic institution in Riyadh, Saudi Arabia, between February and April 2022.

### Participants

**Figure 1:**
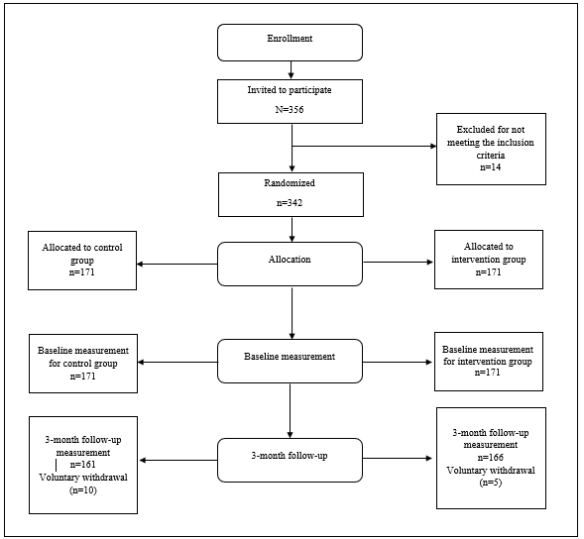
Flowchart of the randomized controlled trial.

To be included in the study, participants had to be (1) 18 years of age or older, (2) a full-time employee, (3) in good health, and (4) own a smartphone. We excluded participants who were younger than 18 years old, pregnant, or had any acute disease or mental illness. Eligible participants received a letter to confirm their approval to participate and were informed about the study protocol. The flowchart of the trial is shown in Figure 1.

### Data Collection

We obtained the email addresses of all full-time employees (1245 people) at the chosen institution. We then randomly selected a preliminary sample of 500, based on the power calculation and accommodating for nonresponse and attrition rates. After randomly selecting the sample of emails, an electronic invitation was sent to the randomly selected employees to participate in the study. The email contained information about informed consent, the study’s background, objectives, procedures, the time required to complete the study, the researcher’s contact information, privacy and confidentiality confirmations, inclusion criteria, and the right to opt-out statement details about the study. Another email was sent as a reminder to ensure a high response rate. Overall, 356 people responded to the invitation email (73% response rate); 342 people met the inclusion criteria and were eligible to participate in the study. After obtaining participant consent and conducting baseline measurements, participants were randomly allocated to groups via computer-generated random numbers in Microsoft Excel for the intervention and control groups with a 1:1 allocation ratio.

### Ethics Approval

The study was approved by the Scientific Ethics Committee of the Saudi Electronic University, Saudi Arabia (SEUREC-22029). All participants provided written informed consent.

### Interventions

#### Intervention Duration

The study was a 2-group RCT conducted from February to April 2022 (3 months), during which 3 visits were conducted. In the first visit, at the end of January, all participants were oriented about the study, and the participants in the intervention group received an orientation session to demonstrate how weekly PA will be measured using the Google Fit: Activity Tracking app (Google) on their smartphones. The second visit was performed 1 week after the first visit to obtain the baseline measurements using a web-based survey to measure the participant’s demographics, weight, and height for participants in both groups. The intervention group had an extra item that asked about the weekly step count obtained from the tracking app. During the second visit, 3 months after the second visit, we obtained the follow-up measurements for both groups. During the whole study period, the participants in the intervention group received 2 text messages each day (1 during the morning hours and 1 during the night hours). Additionally, at the end of each week, participants in the intervention group received weekly instructions to self-monitor their weekly step count obtained from the smartphone app, keep a diary of their weekly steps, and provide any feedback about the intervention. The control group received no text messages for the whole duration of the study.

#### Type of Intervention

Text messages were the primary intervention delivery tool, along with self-monitoring of the weekly step count. The content of the text messages for the intervention group was designed to be concise but effective in promoting PA. The length of each text message averaged 200 characters. The content of each message was modified based on weekly feedback from participants and the improvement of weekly steps. The method for designing the modified text message content over the study period is shown in Table 1. In total, approximately 260 text messages were designed by the authors, who specialized in health education and promotion. The text messages were grounded in behavioral theories, models, and concepts. For instance, the transtheoretical model was used to determine the appropriate text message that aligned with the stage of behavior change for the participants. Additionally, major concepts such as self-efficacy, social support, motivation, and locus of control have been used to improve test-message effectiveness in promoting PA. All prepared text messages were checked for readability and comprehensibility by focus group interviews conducted prior to the initiation of the intervention.

**Table 1:** Methodology for designing the modified text messages content over the study period.

Time point in the study		Description
End of week 1		Participants in the intervention group reported their weekly step count. Authors identified the level of PA^a^ from the baseline measurements.
**Weeks 2 to 12**		Each week, the participant reported their weekly step count and feedback about their progress. The content of the text messages was customized according to participant progress and feedback.
	Week 2	Text messages contained goal setting, action planning, prompts or cues, benefits of PA, and self-efficacy content.
	Weeks 3 to 12	If the goal was achieved, additional challenges, social support, and self-reward were added to the text message content. If the goal was not achieved, problem-solving methods and restructuring of the physical environment were offered.
End of week 13		Participants in the intervention group reported their final week step count.

^a^PA: physical activity.

### Outcome Measures

The study’s primary outcome was the change in average weekly step count for the whole 3-month study duration between the pre- and postmeasurements in each group. The weekly step count was measured by the Google Fit: Activity Tracking app, which applies to most smartphones. The study’s secondary outcome was the difference in the BMI, calculated by self-reported weight and height.

### Sample Size

According to the power calculations, a sample size of 280 (140 participants per group) can be sufficient to detect an anticipated true difference of 2000 mean weekly step count (anticipated SD 3000) between the intervention and control groups with 80% power and a .05 significance level (2-sided). To accommodate missing participants, we adjusted for a dropout rate of 20% in both groups. Power calculations were performed in Stata 17 (StataCorp LLC).

### Statistical Analysis

To estimate the intervention effects between the 2 groups, we followed the intention-to-treat analysis. No observations were lost due to missing data; however, 15 participants requested to drop out of the study. The dropout rates for the intervention and control groups were 5 and 10 participants, respectively. In the initial stage of the analysis, we obtained the descriptive and frequency statistics, means, proportions, and SDs of all variables for the intervention and control groups. We used *t* tests or chi-square tests, as appropriate, to compare the characteristics of the 2 groups. After checking for normality, both the primary and secondary outcomes were normally distributed. To estimate the change in the primary and secondary outcomes between the 2 groups, we performed a linear regression analysis. To account for the occurrence of the regression to the mean, we adjusted for the baseline value of the outcome. Data analyses were performed with SAS version 9.4 (SAS Institute). *P* values less than .05 were considered statistically significant for all tests.

## Results

### Participants

A total of 342 participants were enrolled and randomly allocated to the intervention or control study (1:1 allocation ratio). A total of 327 participants completed the baseline and 3-month follow-up measurements. Table 2 presents baseline participant characteristics for the intervention and control groups. In the first month of the study, 15 participants notified the authors of their voluntary withdrawal from the study. At the follow-up measurement, the number of participants in the intervention and the control group was 166 and 161, respectively. No significant differences were found between the 2 groups at baseline.

**Table 2:** Baseline participant characteristics (N=327).

Variable		Control (n=161)	Intervention (n=166)	*P* value
**Gender, n (%)**				.82
	Male	69 (42.9)	6 (41.6)	
	Female	92 (57.1)	97 (58.4)	
**Age (years), n (%)**				.13
	18-24	45 (28)	55 (33.1)	
	25-34	107 (66.5)	108 (65.1)	
	≥35	9 (5.6)	3 (1.8)	
**Marital status, n (%)**				.91
	Married	59 (36.6)	60 (36.1)	
	Single	97 (63.3)	101 (63.9)	
**Education, n (%)**				.95
	Bachelor or below	151 (93.8)	156 (94)	
	Masters or above	10 (6.2)	10 (6)	
Weight, mean (SD)		69.32 (12.31)	69.14 (11.95)	.89
Height, mean (SD)		164.20 (8.48)	164.07 (8.41)	.87
BMI, mean (SD)		25.68 (3.85)	25.67 (3.86)	.98
Physical activity, mean (SD)		3851.61 (2574.18)	3888.37 (2531.23)	.91

### Outcomes and Estimations

Over the 3-month follow-up period, the average weekly step counts for the intervention group and control group were 4984 steps (SD 2483) and 3853 steps (SD 2590), respectively. No significant within-group differences were detected by age or sex. In the intervention group, participants significantly increased their step count (*P*<.001). In the control group, there were no significant changes in pre- and postintervention step counts. Table 3 presents the means and SDs for PA and BMI for the intervention and control groups. Simple linear regression was used to test if the change in step count was significant between the groups after adjusting for the baseline measure of step count. The overall regression model was statistically significant (*F*=81.15, *P*<.001). The model estimated that the intervention group had a significant increase of 1097 compared to the control group (β=1097, 95% CI 922-1272, *P*<.001) with a large effect size (*d*=1.34; CI 1.10-1.58).

**Table 3:** Physical activity and BMI levels in the intervention and control groups.

Variable		Baseline	Follow-up
**Physical activity (steps/week), mean (SD)**			
	Intervention group (n=166)	3888.37 (2531.23)	4984.94 (2483.54)
	Control group (n=161)	3851.61 (2574.18)	3853.17 (2590.94)
**BMI, mean (SD)**			
	Intervention group (n=166)	25.67 (3.86)	25.09 (3.82)
	Control group (n=161)	25.68 (3.85)	25.69 (3.83)

For the secondary outcome, BMI, the between-groups difference was minimal but significant in average BMI (*P*<.001). In the 3-month follow-up measurements, the intervention group’s average BMI (mean 25.10, SD 3.81) decreased significantly relative to the average BMI of the control group (mean 25.7, SD 3.84). We used simple linear regression to test the significance of the difference between the groups after adjusting for the baseline measure of BMI. Overall, the regression was significant (*F*=72.37, *P*<.001). According to the model, the intervention group had a significant decrease of 0.60 compared to the control group (β=0.60, 95% CI 0.50-0.69, *P*<.001).

## Discussion

### Principal Findings

mHealth and its applications have shown increasing promise in enhancing and maintaining PA levels among the public [32,33]. However, the extent of PA improvements due to mHealth interventions varied substantially depending on the type of intervention or the studied population. To our knowledge, this is the first RCT to examine the effectiveness of tailored text messages combined with self-monitoring in improving PA for healthy adults in the workplace. In our study, we examined the effectiveness of mHealth among adults known to have mostly sedentary computer-based jobs in an academic institution. Our mHealth intervention incorporated several features. First, we used automated randomization to maintain the concealed allocation of participants between the groups and ensure a balance of baseline factors. Second, rather than relying on self-reported measurements of PA, we objectively measured weekly PA by recording weekly step counts using the smartphone app. Third, participants received 2 text messages every day for the whole period of the intervention to effectively maintain engagement levels. Fourth, sent text messages were tailored by experts in the field based on weekly participant feedback. Fifth, we combined text messages with self-monitoring every week to examine their collective effect on PA. Overall, our findings demonstrated a significant difference in both primary (PA) and secondary (BMI) outcomes, which will help inform future interventions and programs.

In addition to the significant improvement in the average weekly step count among the intervention group, the impact of the intervention was encouraging. Our findings showed an increase of 1097 steps/week with a large effect size (*d*=1.34; CI 1.10-1.58) using objective measurement of PA, which drives accurate results relative to self-reported measures [10]. This difference in step count is consistent with recent systematic reviews and meta-analyses of RCTs conducted on various demographics [34-37]. According to recent meta-analysis research, technology-based interventions improved PA with a small to medium effect size [32,33,38]. However, this increase in PA is less than the improvement found in previous research, which revealed an average increase of up to 1566 steps per day [1]. Despite the small improvement in PA in our study, it might have a substantial impact, as it is estimated that an increase of even 1000 steps can result in clinical benefits, including a 10% lower risk of developing metabolic syndrome and a 6% reduction in all-cause mortality [39,40].

Our study examined the effect of using text messages combined with self-monitoring to promote PA in the workplace. All of the study participants were full-time employees of an academic institution, where most of the employees have desk work with minimal PA. Most of the previous RCT studies on promoting PA using mHealth focused on the general population [38], patients with chronic diseases [41], females [42], or the elderly [43]. Studies found that the effect size of mHealth interventions was twice as high in sick or at-risk groups compared to healthy populations [32]. However, mHealth is still effective in all populations [44].

In terms of establishing the difference in PA levels within each group, no significant variations were identified based on the participant’s gender or age group. Such data may seem to contradict the conventional findings, which indicate that men are more active than females and that the younger population is more active than the elderly population [44]. However, research conducted in Saudi Arabia revealed that both men and women are motivated to use mobile interventions to improve their PA [42]. Moreover, in the last decade, the proportion of women who participate in PA has grown, resulting in a difference in the degree of adherence between men and women [43]. In our study, the participants were relatively young. It is possible that the failure to identify a difference between age groups is due to the fact that there is less variation in age groups among participants. Lastly, the within-group analysis was conducted within a short time frame (3 months), and a small sample size may not be sufficient to identify a difference between the participants. More study is needed to evaluate the relationship between age groups, gender, and PA interventions.

Our results indicate the effectiveness of mHealth in improving PA during a 3-month follow-up period. However, the long-term sustainability of the improved impact and the retention of the magnitude of the effect in PA were not investigated. Other RCT studies have evaluated the long-term impact of mHealth interventions and demonstrated a sustained improvement in PA for up to 2 years, with the largest magnitude of improvement in interventions with less than 4 months of follow-up [33]. Such gains tend to remain significant but diminish between 6 and 12 months, with effect sizes decreasing progressively from 0.19 to 0.25 [40]. As a result of the scarcity of long-term studies on the effect of mHealth on PA, further studies, particularly with a longitudinal design, are needed to validate the long-term effects (greater than 2 years) of mHealth interventions on PA.

Our intervention was relatively cost-effective considering the effect size of the PA level and the anticipated benefits of maintaining an active lifestyle. The automated text message dissemination to the participants required low overhead, with a calculated cost of Aus $0.10 (US $0.0673) per message (Aus $18 [US $12.11] per participant). Other studies reported a range of Aus $0.10 (US $0.0673) to Aus $0.30 (US $0.201) per message [45,46] and estimated the cost of a national intervention to be Aus $22.37 (US $15.05) per participant (Aus $2693 [US $1812.3] per quality-adjusted life year) [47]. This highlights the feasibility of scaling the intervention to the population level, as text messages do not require a smartphone and are agnostic to all phone operating systems [45]. Implementing a large-scale intervention is a promising and far-reaching approach since adopting an active lifestyle is linked to a decreased demand for primary and secondary health care services [47].

As part of our intervention, we used a combined approach to promote PA through text messages and advised participants to self-monitor their weekly step count. Setting goals on a weekly or biweekly basis has been found to be more effective than setting daily goals [34,48]. The use of text messages in conjunction with self-monitoring has shown promise since multiple studies have established that multicomponent programs have a greater impact than single-component interventions [32]. Specifically, combining tailored text messages with tracking PA performance was more effective than using self-monitoring alone and web-based interventions [48-52].

The baseline level of PA was lower than the minimum recommended level of PA associated with health benefits [53,54]. The WHO recommends 10,000 steps per day and defines sedentary living as less than 5000 steps per day [55]. The significant difference observed in our study can be explained by our sample’s lower PA baseline; consequently, there was a greater chance for improvement. Several studies have identified a link between baseline PA levels and mHealth intervention effectiveness [54,56-59]. Additionally, the implementation of preventative measures such as social distancing and lockdown restrictions during the COVID-19 pandemic between 2020 and 2021 might reduce the level of PA in the postpandemic period. This was observed in large national studies that showed a decrease from 21.5% to 10.90% in moderate PA and reported that only 30% of people had regained the level of PA before the pandemic [60], which is concerning and calls for more research to evaluate the trends at the population level and to explore potential solutions.

In examining the secondary outcome, we found that participants effectively reduced their BMI significantly during the intervention, which is consistent with related research. For instance, a large systematic review study found a significant association between BMI and systolic blood pressure [61]. Another study estimated that over an average of 6 years, an increase of 2000 steps/day and a decrease in BMI were associated with a 10% relative decrease in the incidence of CVD [62]. Additionally, maintaining recommended levels of BMI over the longer term produced a positive circulating metabolome and diminished inflammation markers, which reduced the risk of various non-CVD illnesses such as cancer, dementia, depression, and all-cause mortality [63,64].

### Limitations

Despite the significant findings and implications of this study, there are some limitations. First, the study has a short duration of 3 months, which could not be prolonged due to administrative constraints. Future studies should design longer interventions, preferably more than 6 months. Second, although the intervention’s effect was substantial enough to identify significant differences between our research groups, the results cannot be generalized due to the limited sample size. Third, we were not able to evaluate the effectiveness of the message’s type and content. Therefore, we could not determine from this study whether the everyday comments, reminders, or educational-motivational messages were specifically accountable for the outcomes of the study.

### Conclusions

In conclusion, this study offers preliminary evidence for the use of tailored text messages and self-monitoring interventions to enhance PA in the workplace. Particularly, our research demonstrates that tailored text messages and self-monitoring interventions can improve PA and reduce BMI and hence complement current efforts to improve public health. In future research, longer interventions in different settings with a larger population may be used to further investigate the impact of tailored text messages and self-monitoring. The text messages used in this study were modified based on weekly participant responses, which has been demonstrated to be more successful than untailored text messages. However, further research must investigate the use of instant feedback from participants, such as evaluating the efficacy of each message, to provide more reliable and effective text message customization based on individual needs. Moreover, future research should compare the effectiveness of different types and doses of messages, as well as their combinations, to discover the best mechanisms and conceptual frameworks. With the use of objective measurement, we were able to determine the total number of steps taken in a given period of time, which was our primary outcome. Future research can evaluate the effect of SMS text messaging on other PA measurements (eg, measures of intensity, differentiating between habitual and merely recent activities, and including leisure and nonleisure activities). Finally, during the course of the 3 months, we observed a negative correlation between BMI and PA and a substantial change in both. However, the influence of nutrition has not been studied. Future studies may investigate the effects of diet and any relationships between PA, BMI, and diet. Given the encouraging findings of this study, tailored text messages and self-monitoring treatments could be successful in enhancing existing workplace interventions aimed at raising PA levels, which, given the low cost and burden of intervention, might have a broad influence on public health.

## Appendix

Multimedia Appendix 1 CONSORT e-Health Checklist.

## Abbreviations

mHealth: mobile health
PA: physical activity
RCT: randomized controlled trial
WHO: World Health Organization
